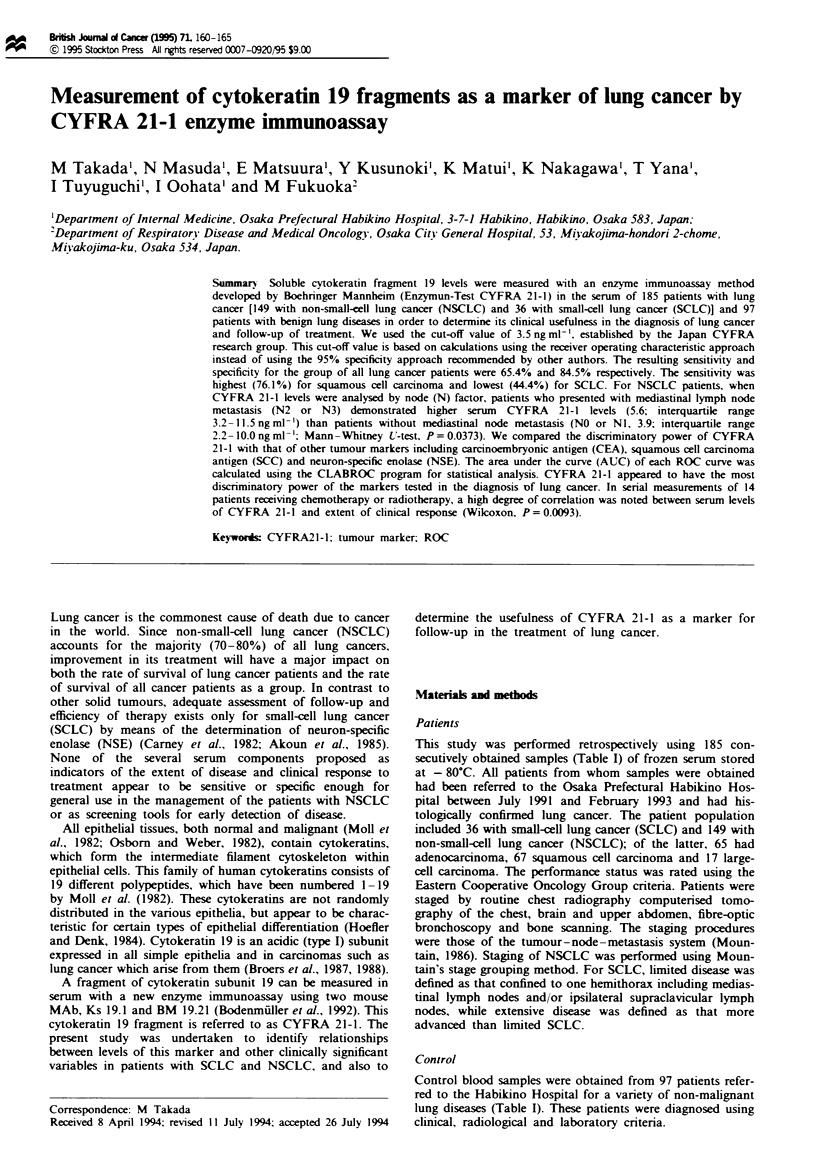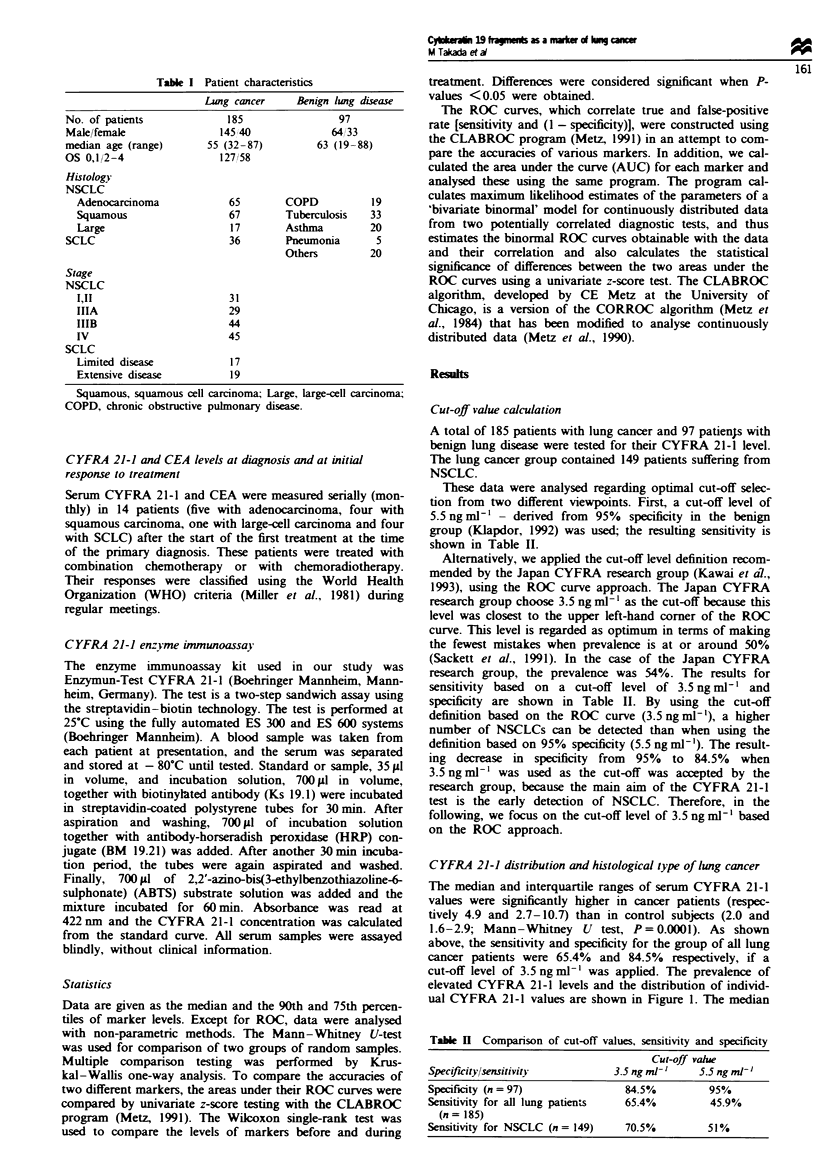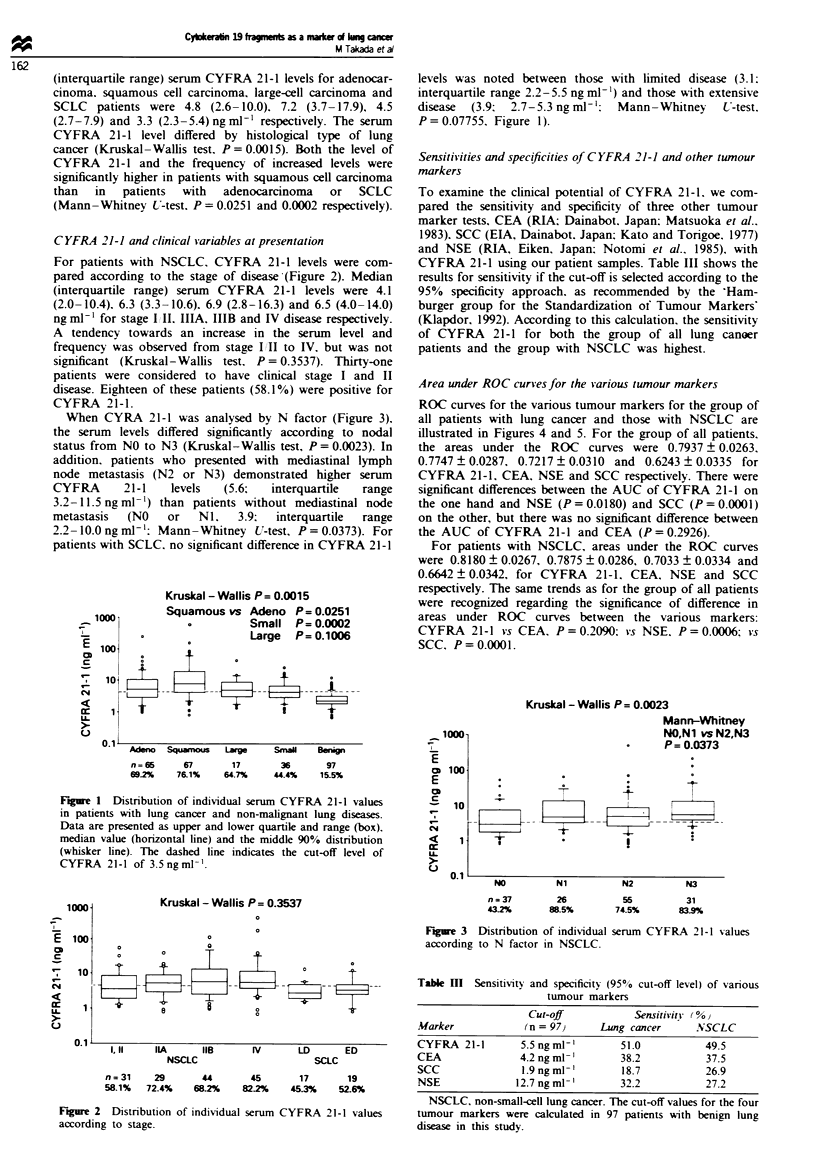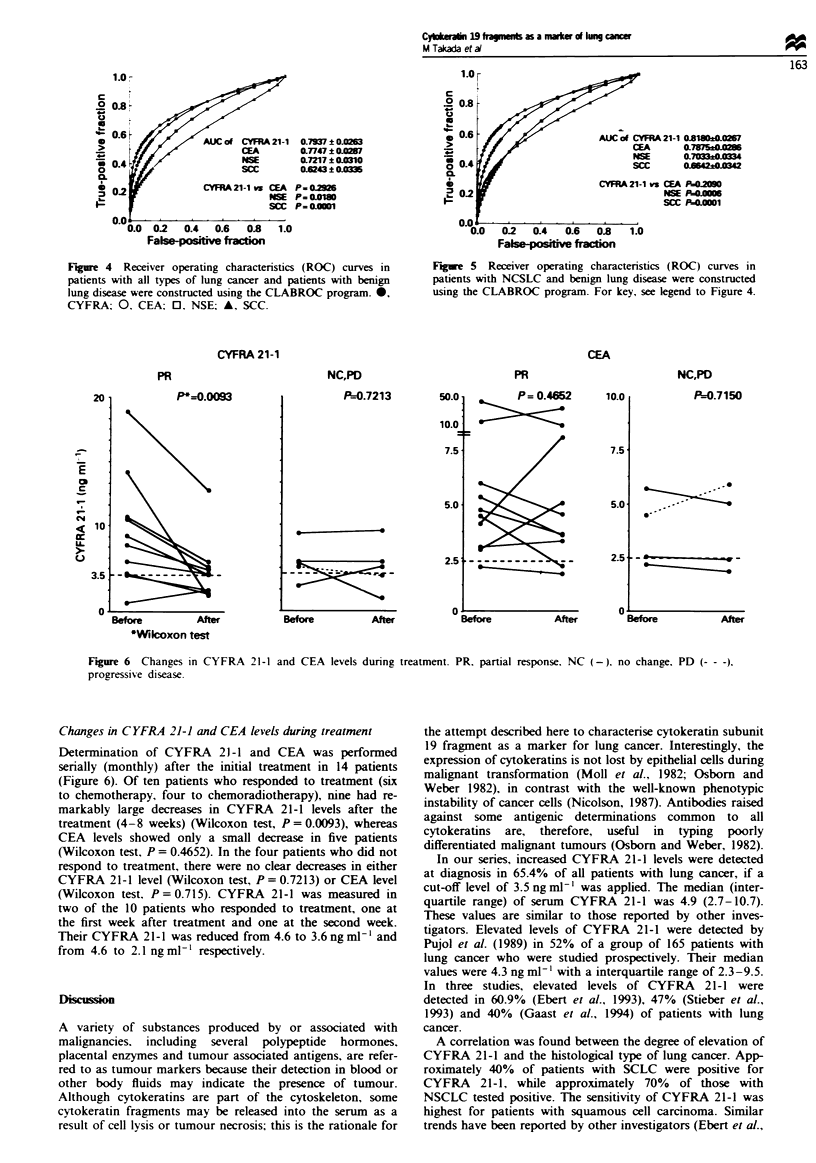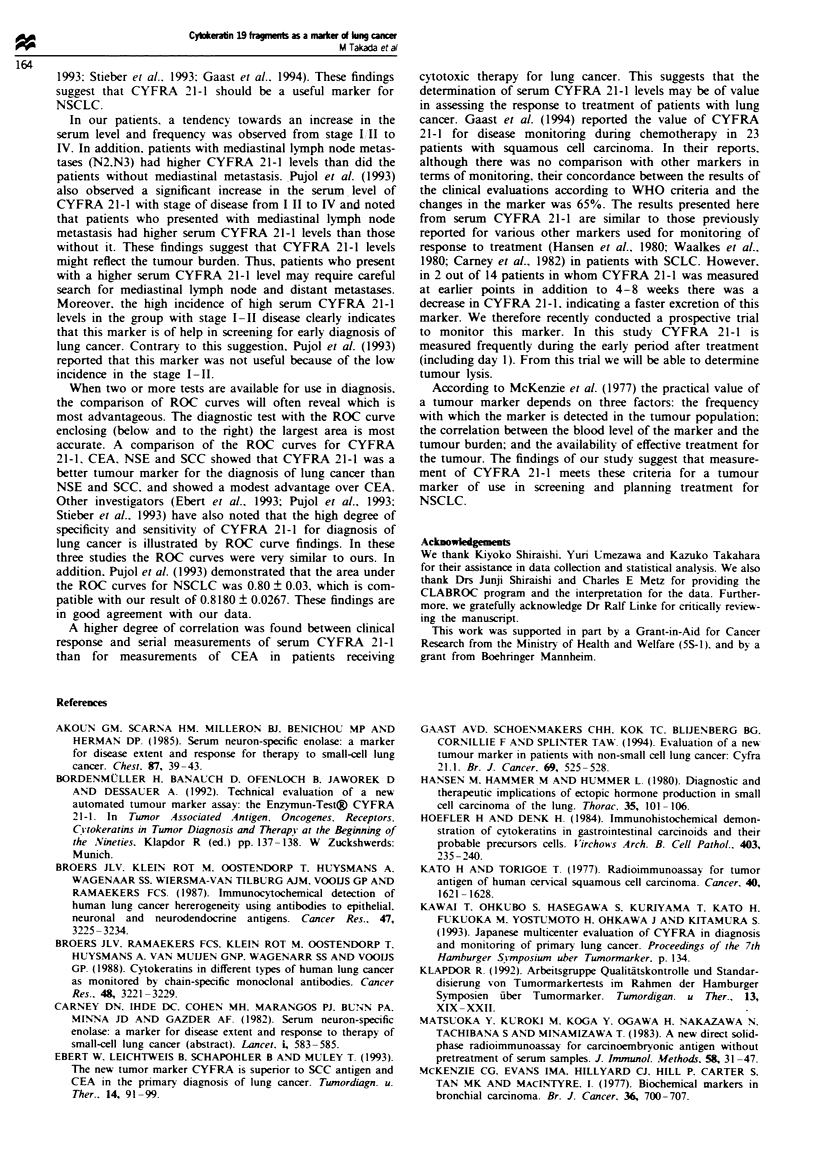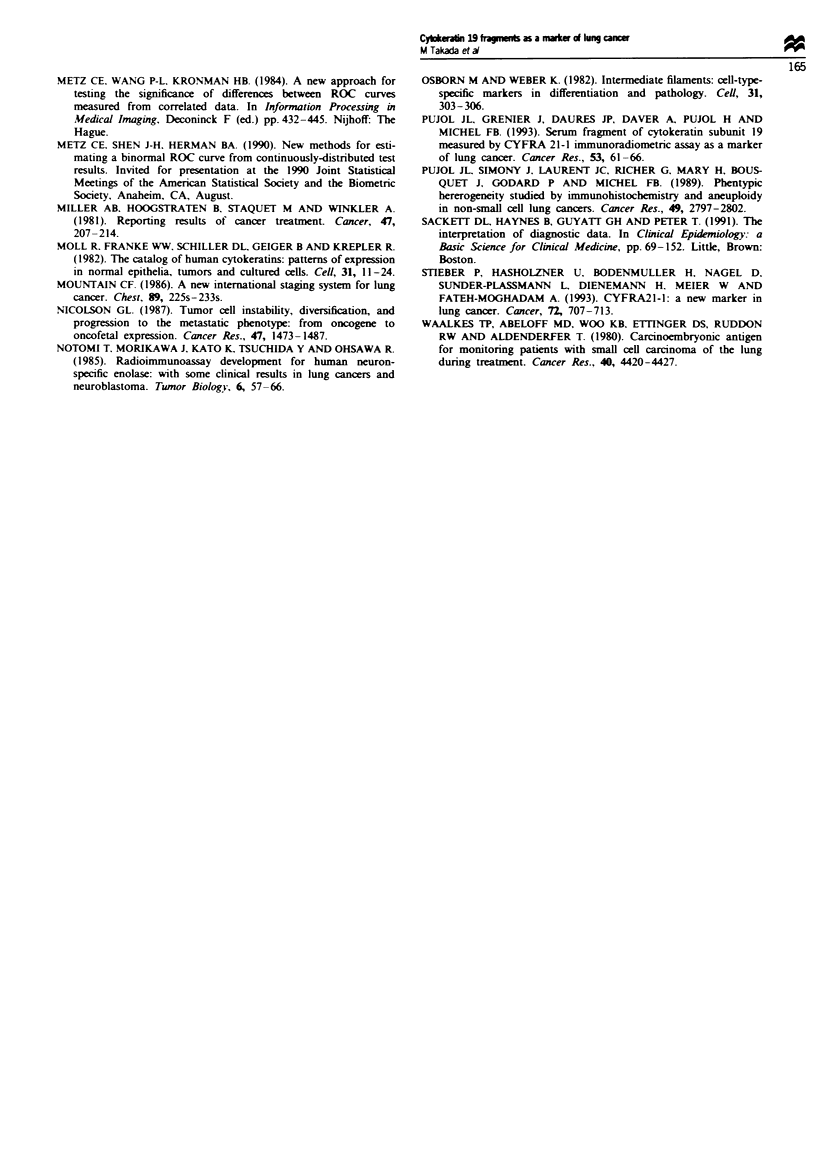# Measurement of cytokeratin 19 fragments as a marker of lung cancer by CYFRA 21-1 enzyme immunoassay.

**DOI:** 10.1038/bjc.1995.33

**Published:** 1995-01

**Authors:** M. Takada, N. Masuda, E. Matsuura, Y. Kusunoki, K. Matui, K. Nakagawa, T. Yana, I. Tuyuguchi, I. Oohata, M. Fukuoka

**Affiliations:** Department of Internal Medicine, Osaka Prefectural Habikino Hospital, Japan.

## Abstract

Soluble cytokeratin fragment 19 levels were measured with an enzyme immunoassay method developed by Boehringer Mannheim (Enzymun-Test CYFRA 21-1) in the serum of 185 patients with lung cancer [149 with non-small-cell lung cancer (NSCLC) and 36 with small-cell lung cancer (SCLC)] and 97 patients with benign lung diseases in order to determine its clinical usefulness in the diagnosis of lung cancer and follow-up of treatment. We used the cut-off value of 3.5 ng ml-1, established by the Japan CYFRA research group. This cut-off value is based on calculations using the receiver operating characteristic approach instead of using the 95% specificity approach recommended by other authors. The resulting sensitivity and specificity for the group of all lung cancer patients were 65.4% and 84.5% respectively. The sensitivity was highest (76.1%) for squamous cell carcinoma and lowest (44.4%) for SCLC. For NSCLC patients, when CYFRA 21-1 levels were analysed by node (N) factor, patients who presented with mediastinal lymph node metastasis (N2 or N3) demonstrated higher serum CYFRA 21-1 levels (5.6; interquartile range 3.2-11.5 ng ml-1) than patients without mediastinal node metastasis (N0 or N1, 3.9; interquartile range 2.2-10.0 ng ml-1; Mann-Whitney U-test, P = 0.0373). We compared the discriminatory power of CYFRA 21-1 with that of other tumour markers including carcinoembryonic antigen (CEA), squamous cell carcinoma antigen (SCC) and neuron-specific enolase (NSE). The area under the curve (AUC) of each ROC curve was calculated using the CLABROC program for statistical analysis. CYFRA 21-1 appeared to have the most discriminatory power of the markers tested in the diagnosis of lung cancer. In serial measurements of 14 patients receiving chemotherapy or radiotherapy, a high degree of correlation was noted between serum levels of CYFRA 21-1 and extent of clinical response (Wilcoxon, P = 0.0093).


British Journal of Cancer (1995) 71, 160-165

© 1995 Stockton Press All rights reserved 0007-0920/95 $9.00

### Measurement of cytokeratin 19 fragments as a marker of lung cancer by CYFRA 21-1 enzyme immunoassay

M Takada[1], N Masuda[1], E Matsuura[1], Y Kusunoki[1], K Matui[1], K Nakagawa[1], T Yana[1], I Tuyuguchi[1], I Oohata[1] and M Fukuoka[2]

[1]*Department of Internal Medicine, Osaka Prefectural Habikino Hospital, 3-7-1 Habikino, Habikino, Osaka 583, Japan;* [2]*Department of Respiratory Disease and Medical Oncology, Osaka City General Hospital, 53, Miyakojima-hondori 2-chome, Miyakojima-ku, Osaka 534, Japan.*

**Summary** Soluble cytokeratin fragment 19 levels were measured with an enzyme immunoassay method developed by Boehringer Mannheim (Enzymun-Test CYFRA 21-1) in the serum of 185 patients with lung cancer [149 with non-small-cell lung cancer (NSCLC) and 36 with small-cell lung cancer (SCLC)] and 97 patients with benign lung diseases in order to determine its clinical usefulness in the diagnosis of lung cancer and follow-up of treatment. We used the cut-off value of 3.5 ng ml$^{-1}$, established by the Japan CYFRA research group. This cut-off value is based on calculations using the receiver operating characteristic approach instead of using the 95% specificity approach recommended by other authors. The resulting sensitivity and specificity for the group of all lung cancer patients were 65.4% and 84.5% respectively. The sensitivity was highest (76.1%) for squamous cell carcinoma and lowest (44.4%) for SCLC. For NSCLC patients, when CYFRA 21-1 levels were analysed by node (N) factor, patients who presented with mediastinal lymph node metastasis (N2 or N3) demonstrated higher serum CYFRA 21-1 levels (5.6; interquartile range 3.2-11.5 ng ml$^{-1}$) than patients without mediastinal node metastasis (N0 or N1, 3.9; interquartile range 2.2-10.0 ng ml$^{-1}$; Mann-Whitney *U*-test, $P = 0.0373$). We compared the discriminatory power of CYFRA 21-1 with that of other tumour markers including carcinoembryonic antigen (CEA), squamous cell carcinoma antigen (SCC) and neuron-specific enolase (NSE). The area under the curve (AUC) of each ROC curve was calculated using the CLABROC program for statistical analysis. CYFRA 21-1 appeared to have the most discriminatory power of the markers tested in the diagnosis of lung cancer. In serial measurements of 14 patients receiving chemotherapy or radiotherapy, a high degree of correlation was noted between serum levels of CYFRA 21-1 and extent of clinical response (Wilcoxon, $P = 0.0093$).

**Keywords:** CYFRA21-1; tumour marker; ROC

Lung cancer is the commonest cause of death due to cancer in the world. Since non-small-cell lung cancer (NSCLC) accounts for the majority (70-80%) of all lung cancers, improvement in its treatment will have a major impact on both the rate of survival of lung cancer patients and the rate of survival of all cancer patients as a group. In contrast to other solid tumours, adequate assessment of follow-up and efficiency of therapy exists only for small-cell lung cancer (SCLC) by means of the determination of neuron-specific enolase (NSE) (Carney *et al.*, 1982; Akoun *et al.*, 1985). None of the several serum components proposed as indicators of the extent of disease and clinical response to treatment appear to be sensitive or specific enough for general use in the management of the patients with NSCLC or as screening tools for early detection of disease.

All epithelial tissues, both normal and malignant (Moll *et al.*, 1982; Osborn and Weber, 1982), contain cytokeratins, which form the intermediate filament cytoskeleton within epithelial cells. This family of human cytokeratins consists of 19 different polypeptides, which have been numbered 1-19 by Moll *et al.* (1982). These cytokeratins are not randomly distributed in the various epithelia, but appear to be characteristic for certain types of epithelial differentiation (Hoefler and Denk, 1984). Cytokeratin 19 is an acidic (type I) subunit expressed in all simple epithelia and in carcinomas such as lung cancer which arise from them (Broers *et al.*, 1987, 1988).

A fragment of cytokeratin subunit 19 can be measured in serum with a new enzyme immunoassay using two mouse MAb, Ks 19.1 and BM 19.21 (Bodenmüller *et al.*, 1992). This cytokeratin 19 fragment is referred to as CYFRA 21-1. The present study was undertaken to identify relationships between levels of this marker and other clinically significant variables in patients with SCLC and NSCLC, and also to determine the usefulness of CYFRA 21-1 as a marker for follow-up in the treatment of lung cancer.

Correspondence: M Takada
Received 8 April 1994; revised 11 July 1994; accepted 26 July 1994

### Materials and methods

### *Patients*

This study was performed retrospectively using 185 consecutively obtained samples (Table I) of frozen serum stored at −80°C. All patients from whom samples were obtained had been referred to the Osaka Prefectural Habikino Hospital between July 1991 and February 1993 and had histologically confirmed lung cancer. The patient population included 36 with small-cell lung cancer (SCLC) and 149 with non-small-cell lung cancer (NSCLC); of the latter, 65 had adenocarcinoma, 67 squamous cell carcinoma and 17 large-cell carcinoma. The performance status was rated using the Eastern Cooperative Oncology Group criteria. Patients were staged by routine chest radiography computerised tomography of the chest, brain and upper abdomen, fibre-optic bronchoscopy and bone scanning. The staging procedures were those of the tumour-node-metastasis system (Mountain, 1986). Staging of NSCLC was performed using Mountain's stage grouping method. For SCLC, limited disease was defined as that confined to one hemithorax including mediastinal lymph nodes and/or ipsilateral supraclavicular lymph nodes, while extensive disease was defined as that more advanced than limited SCLC.

### *Control*

Control blood samples were obtained from 97 patients referred to the Habikino Hospital for a variety of non-malignant lung diseases (Table I). These patients were diagnosed using clinical, radiological and laboratory criteria.

**Table I** Patient characteristics

| | *Lung cancer* | *Benign lung disease* | |
|---|---|---|---|
| No. of patients | 185 | 97 | |
| Male/female | 145/40 | 64/33 | |
| median age (range) | 55 (32–87) | 63 (19–88) | |
| OS 0,1/2–4 | 127/58 | | |
| *Histology* | | | |
| NSCLC | | | |
| Adenocarcinoma | 65 | COPD | 19 |
| Squamous | 67 | Tuberculosis | 33 |
| Large | 17 | Asthma | 20 |
| SCLC | 36 | Pneumonia | 5 |
| | | Others | 20 |
| *Stage* | | | |
| NSCLC | | | |
| I,II | 31 | | |
| IIIA | 29 | | |
| IIIB | 44 | | |
| IV | 45 | | |
| SCLC | | | |
| Limited disease | 17 | | |
| Extensive disease | 19 | | |

Squamous, squamous cell carcinoma; Large, large-cell carcinoma; COPD, chronic obstructive pulmonary disease.

### *CYFRA 21-1 and CEA levels at diagnosis and at initial response to treatment*

Serum CYFRA 21-1 and CEA were measured serially (monthly) in 14 patients (five with adenocarcinoma, four with squamous carcinoma, one with large-cell carcinoma and four with SCLC) after the start of the first treatment at the time of the primary diagnosis. These patients were treated with combination chemotherapy or with chemoradiotherapy. Their responses were classified using the World Health Organization (WHO) criteria (Miller *et al.*, 1981) during regular meetings.

### *CYFRA 21-1 enzyme immunoassay*

The enzyme immunoassay kit used in our study was Enzymun-Test CYFRA 21-1 (Boehringer Mannheim, Mannheim, Germany). The test is a two-step sandwich assay using the streptavidin–biotin technology. The test is performed at 25°C using the fully automated ES 300 and ES 600 systems (Boehringer Mannheim). A blood sample was taken from each patient at presentation, and the serum was separated and stored at − 80°C until tested. Standard or sample, 35 µl in volume, and incubation solution, 700 µl in volume, together with biotinylated antibody (Ks 19.1) were incubated in streptavidin-coated polystyrene tubes for 30 min. After aspiration and washing, 700 µl of incubation solution together with antibody-horseradish peroxidase (HRP) conjugate (BM 19.21) was added. After another 30 min incubation period, the tubes were again aspirated and washed. Finally, 700 µl of 2,2′-azino-bis(3-ethylbenzothiazoline-6-sulphonate) (ABTS) substrate solution was added and the mixture incubated for 60 min. Absorbance was read at 422 nm and the CYFRA 21-1 concentration was calculated from the standard curve. All serum samples were assayed blindly, without clinical information.

### *Statistics*

Data are given as the median and the 90th and 75th percentiles of marker levels. Except for ROC, data were analysed with non-parametric methods. The Mann–Whitney *U*-test was used for comparison of two groups of random samples. Multiple comparison testing was performed by Kruskal–Wallis one-way analysis. To compare the accuracies of two different markers, the areas under their ROC curves were compared by univariate *z*-score testing with the CLABROC program (Metz, 1991). The Wilcoxon single-rank test was used to compare the levels of markers before and during treatment. Differences were considered significant when *P*-values <0.05 were obtained.

The ROC curves, which correlate true and false-positive rate [sensitivity and (1 − specificity)], were constructed using the CLABROC program (Metz, 1991) in an attempt to compare the accuracies of various markers. In addition, we calculated the area under the curve (AUC) for each marker and analysed these using the same program. The program calculates maximum likelihood estimates of the parameters of a 'bivariate binormal' model for continuously distributed data from two potentially correlated diagnostic tests, and thus estimates the binormal ROC curves obtainable with the data and their correlation and also calculates the statistical significance of differences between the two areas under the ROC curves using a univariate *z*-score test. The CLABROC algorithm, developed by CE Metz at the University of Chicago, is a version of the CORROC algorithm (Metz *et al.*, 1984) that has been modified to analyse continuously distributed data (Metz *et al.*, 1990).

### Results

### *Cut-off value calculation*

A total of 185 patients with lung cancer and 97 patients with benign lung disease were tested for their CYFRA 21-1 level. The lung cancer group contained 149 patients suffering from NSCLC.

These data were analysed regarding optimal cut-off selection from two different viewpoints. First, a cut-off level of 5.5 ng $ml^{-1}$ – derived from 95% specificity in the benign group (Klapdor, 1992) was used; the resulting sensitivity is shown in Table II.

Alternatively, we applied the cut-off level definition recommended by the Japan CYFRA research group (Kawai *et al.*, 1993), using the ROC curve approach. The Japan CYFRA research group choose 3.5 ng $ml^{-1}$ as the cut-off because this level was closest to the upper left-hand corner of the ROC curve. This level is regarded as optimum in terms of making the fewest mistakes when prevalence is at or around 50% (Sackett *et al.*, 1991). In the case of the Japan CYFRA research group, the prevalence was 54%. The results for sensitivity based on a cut-off level of 3.5 ng $ml^{-1}$ and specificity are shown in Table II. By using the cut-off definition based on the ROC curve (3.5 ng $ml^{-1}$), a higher number of NSCLCs can be detected than when using the definition based on 95% specificity (5.5 ng $ml^{-1}$). The resulting decrease in specificity from 95% to 84.5% when 3.5 ng $ml^{-1}$ was used as the cut-off was accepted by the research group, because the main aim of the CYFRA 21-1 test is the early detection of NSCLC. Therefore, in the following, we focus on the cut-off level of 3.5 ng $ml^{-1}$ based on the ROC approach.

### *CYFRA 21-1 distribution and histological type of lung cancer*

The median and interquartile ranges of serum CYFRA 21-1 values were significantly higher in cancer patients (respectively 4.9 and 2.7–10.7) than in control subjects (2.0 and 1.6–2.9; Mann–Whitney *U* test, $P = 0.0001$). As shown above, the sensitivity and specificity for the group of all lung cancer patients were 65.4% and 84.5% respectively, if a cut-off level of 3.5 ng $ml^{-1}$ was applied. The prevalence of elevated CYFRA 21-1 levels and the distribution of individual CYFRA 21-1 values are shown in Figure 1. The median

**Table II** Comparison of cut-off values, sensitivity and specificity

| | *Cut-off value* | |
|---|---|---|
| *Specificity/sensitivity* | *3.5 ng $ml^{-1}$* | *5.5 ng $ml^{-1}$* |
| Specificity ($n = 97$) | 84.5% | 95% |
| Sensitivity for all lung patients ($n = 185$) | 65.4% | 45.9% |
| Sensitivity for NSCLC ($n = 149$) | 70.5% | 51% |

(interquartile range) serum CYFRA 21-1 levels for adenocarcinoma, squamous cell carcinoma, large-cell carcinoma and SCLC patients were 4.8 (2.6–10.0), 7.2 (3.7–17.9), 4.5 (2.7–7.9) and 3.3 (2.3–5.4) ng $ml^{-1}$ respectively. The serum CYFRA 21-1 level differed by histological type of lung cancer (Kruskal–Wallis test, $P = 0.0015$). Both the level of CYFRA 21-1 and the frequency of increased levels were significantly higher in patients with squamous cell carcinoma than in patients with adenocarcinoma or SCLC (Mann–Whitney $U$-test, $P = 0.0251$ and 0.0002 respectively).

### *CYFRA 21-1 and clinical variables at presentation*

For patients with NSCLC, CYFRA 21-1 levels were compared according to the stage of disease (Figure 2). Median (interquartile range) serum CYFRA 21-1 levels were 4.1 (2.0–10.4), 6.3 (3.3–10.6), 6.9 (2.8–16.3) and 6.5 (4.0–14.0) ng $ml^{-1}$ for stage I/II, IIIA, IIIB and IV disease respectively. A tendency towards an increase in the serum level and frequency was observed from stage I/II to IV, but was not significant (Kruskal–Wallis test, $P = 0.3537$). Thirty-one patients were considered to have clinical stage I and II disease. Eighteen of these patients (58.1%) were positive for CYFRA 21-1.

When CYRA 21-1 was analysed by N factor (Figure 3), the serum levels differed significantly according to nodal status from N0 to N3 (Kruskal–Wallis test, $P = 0.0023$). In addition, patients who presented with mediastinal lymph node metastasis (N2 or N3) demonstrated higher serum CYFRA 21-1 levels (5.6; interquartile range 3.2–11.5 ng $ml^{-1}$) than patients without mediastinal node metastasis (N0 or N1, 3.9; interquartile range 2.2–10.0 ng $ml^{-1}$; Mann–Whitney $U$-test, $P = 0.0373$). For patients with SCLC, no significant difference in CYFRA 21-1 levels was noted between those with limited disease (3.1; interquartile range 2.2–5.5 ng $ml^{-1}$) and those with extensive disease (3.9; 2.7–5.3 ng $ml^{-1}$; Mann–Whitney $U$-test, $P = 0.07755$, Figure 1).

**Figure 1** Distribution of individual serum CYFRA 21-1 values in patients with lung cancer and non-malignant lung diseases. Data are presented as upper and lower quartile and range (box), median value (horizontal line) and the middle 90% distribution (whisker line). The dashed line indicates the cut-off level of CYFRA 21-1 of 3.5 ng $ml^{-1}$.

**Figure 2** Distribution of individual serum CYFRA 21-1 values according to stage.

### *Sensitivities and specificities of CYFRA 21-1 and other tumour markers*

To examine the clinical potential of CYFRA 21-1, we compared the sensitivity and specificity of three other tumour marker tests, CEA (RIA; Dainabot, Japan; Matsuoka *et al.*, 1983), SCC (EIA, Dainabot, Japan; Kato and Torigoe, 1977) and NSE (RIA, Eiken, Japan; Notomi *et al.*, 1985), with CYFRA 21-1 using our patient samples. Table III shows the results for sensitivity if the cut-off is selected according to the 95% specificity approach, as recommended by the 'Hamburger group for the Standardization of Tumour Markers' (Klapdor, 1992). According to this calculation, the sensitivity of CYFRA 21-1 for both the group of all lung cancer patients and the group with NSCLC was highest.

### *Area under ROC curves for the various tumour markers*

ROC curves for the various tumour markers for the group of all patients with lung cancer and those with NSCLC are illustrated in Figures 4 and 5. For the group of all patients, the areas under the ROC curves were $0.7937 \pm 0.0263$, $0.7747 \pm 0.0287$, $0.7217 \pm 0.0310$ and $0.6243 \pm 0.0335$ for CYFRA 21-1, CEA, NSE and SCC respectively. There were significant differences between the AUC of CYFRA 21-1 on the one hand and NSE ($P = 0.0180$) and SCC ($P = 0.0001$) on the other, but there was no significant difference between the AUC of CYFRA 21-1 and CEA ($P = 0.2926$).

For patients with NSCLC, areas under the ROC curves were $0.8180 \pm 0.0267$, $0.7875 \pm 0.0286$, $0.7033 \pm 0.0334$ and $0.6642 \pm 0.0342$, for CYFRA 21-1, CEA, NSE and SCC respectively. The same trends as for the group of all patients were recognized regarding the significance of difference in areas under ROC curves between the various markers: CYFRA 21-1 *vs* CEA, $P = 0.2090$; *vs* NSE, $P = 0.0006$; *vs* SCC, $P = 0.0001$.

**Figure 3** Distribution of individual serum CYFRA 21-1 values according to N factor in NSCLC.

**Table III** Sensitivity and specificity (95% cut-off level) of various tumour markers

| Marker | Cut-off (n = 97) | Sensitivity (%) Lung cancer | Sensitivity (%) NSCLC |
|---|---|---|---|
| CYFRA 21-1 | 5.5 ng $ml^{-1}$ | 51.0 | 49.5 |
| CEA | 4.2 ng $ml^{-1}$ | 38.2 | 37.5 |
| SCC | 1.9 ng $ml^{-1}$ | 18.7 | 26.9 |
| NSE | 12.7 ng $ml^{-1}$ | 32.2 | 27.2 |

NSCLC, non-small-cell lung cancer. The cut-off values for the four tumour markers were calculated in 97 patients with benign lung disease in this study.

**Figure 4** Receiver operating characteristics (ROC) curves in patients with all types of lung cancer and patients with benign lung disease were constructed using the CLABROC program. ●, CYFRA; ○, CEA; □, NSE; ▲, SCC.

**Figure 5** Receiver operating characteristics (ROC) curves in patients with NCSLC and benign lung disease were constructed using the CLABROC program. For key, see legend to Figure 4.

**Figure 6** Changes in CYFRA 21-1 and CEA levels during treatment. PR, partial response, NC (—), no change, PD (- - -), progressive disease.

### *Changes in CYFRA 21-1 and CEA levels during treatment*

Determination of CYFRA 21-1 and CEA was performed serially (monthly) after the initial treatment in 14 patients (Figure 6). Of ten patients who responded to treatment (six to chemotherapy, four to chemoradiotherapy), nine had remarkably large decreases in CYFRA 21-1 levels after the treatment (4–8 weeks) (Wilcoxon test, $P = 0.0093$), whereas CEA levels showed only a small decrease in five patients (Wilcoxon test, $P = 0.4652$). In the four patients who did not respond to treatment, there were no clear decreases in either CYFRA 21-1 level (Wilcoxon test, $P = 0.7213$) or CEA level (Wilcoxon test, $P = 0.715$). CYFRA 21-1 was measured in two of the 10 patients who responded to treatment, one at the first week after treatment and one at the second week. Their CYFRA 21-1 was reduced from 4.6 to 3.6 ng $ml^{-1}$ and from 4.6 to 2.1 ng $ml^{-1}$ respectively.

### Discussion

A variety of substances produced by or associated with malignancies, including several polypeptide hormones, placental enzymes and tumour associated antigens, are referred to as tumour markers because their detection in blood or other body fluids may indicate the presence of tumour. Although cytokeratins are part of the cytoskeleton, some cytokeratin fragments may be released into the serum as a result of cell lysis or tumour necrosis; this is the rationale for the attempt described here to characterise cytokeratin subunit 19 fragment as a marker for lung cancer. Interestingly, the expression of cytokeratins is not lost by epithelial cells during malignant transformation (Moll *et al.*, 1982; Osborn and Weber 1982), in contrast with the well-known phenotypic instability of cancer cells (Nicolson, 1987). Antibodies raised against some antigenic determinations common to all cytokeratins are, therefore, useful in typing poorly differentiated malignant tumours (Osborn and Weber, 1982).

In our series, increased CYFRA 21-1 levels were detected at diagnosis in 65.4% of all patients with lung cancer, if a cut-off level of 3.5 ng $ml^{-1}$ was applied. The median (interquartile range) of serum CYFRA 21-1 was 4.9 (2.7–10.7). These values are similar to those reported by other investigators. Elevated levels of CYFRA 21-1 were detected by Pujol *et al.* (1989) in 52% of a group of 165 patients with lung cancer who were studied prospectively. Their median values were 4.3 ng $ml^{-1}$ with a interquartile range of 2.3–9.5. In three studies, elevated levels of CYFRA 21-1 were detected in 60.9% (Ebert *et al.*, 1993), 47% (Stieber *et al.*, 1993) and 40% (Gaast *et al.*, 1994) of patients with lung cancer.

A correlation was found between the degree of elevation of CYFRA 21-1 and the histological type of lung cancer. Approximately 40% of patients with SCLC were positive for CYFRA 21-1, while approximately 70% of those with NSCLC tested positive. The sensitivity of CYFRA 21-1 was highest for patients with squamous cell carcinoma. Similar trends have been reported by other investigators (Ebert *et al.*,

1993; Stieber et al., 1993; Gaast et al., 1994). These findings suggest that CYFRA 21-1 should be a useful marker for NSCLC.

In our patients, a tendency towards an increase in the serum level and frequency was observed from stage I/II to IV. In addition, patients with mediastinal lymph node metastases (N2,N3) had higher CYFRA 21-1 levels than did the patients without mediastinal metastasis. Pujol et al. (1993) also observed a significant increase in the serum level of CYFRA 21-1 with stage of disease from I/II to IV and noted that patients who presented with mediastinal lymph node metastasis had higher serum CYFRA 21-1 levels than those without it. These findings suggest that CYFRA 21-1 levels might reflect the tumour burden. Thus, patients who present with a higher serum CYFRA 21-1 level may require careful search for mediastinal lymph node and distant metastases. Moreover, the high incidence of high serum CYFRA 21-1 levels in the group with stage I–II disease clearly indicates that this marker is of help in screening for early diagnosis of lung cancer. Contrary to this suggestion, Pujol et al. (1993) reported that this marker was not useful because of the low incidence in the stage I–II.

When two or more tests are available for use in diagnosis, the comparison of ROC curves will often reveal which is most advantageous. The diagnostic test with the ROC curve enclosing (below and to the right) the largest area is most accurate. A comparison of the ROC curves for CYFRA 21-1, CEA, NSE and SCC showed that CYFRA 21-1 was a better tumour marker for the diagnosis of lung cancer than NSE and SCC, and showed a modest advantage over CEA. Other investigators (Ebert et al., 1993; Pujol et al., 1993; Stieber et al., 1993) have also noted that the high degree of specificity and sensitivity of CYFRA 21-1 for diagnosis of lung cancer is illustrated by ROC curve findings. In these three studies the ROC curves were very similar to ours. In addition, Pujol et al. (1993) demonstrated that the area under the ROC curves for NSCLC was $0.80 \pm 0.03$, which is compatible with our result of $0.8180 \pm 0.0267$. These findings are in good agreement with our data.

A higher degree of correlation was found between clinical response and serial measurements of serum CYFRA 21-1 than for measurements of CEA in patients receiving cytotoxic therapy for lung cancer. This suggests that the determination of serum CYFRA 21-1 levels may be of value in assessing the response to treatment of patients with lung cancer. Gaast et al. (1994) reported the value of CYFRA 21-1 for disease monitoring during chemotherapy in 23 patients with squamous cell carcinoma. In their reports, although there was no comparison with other markers in terms of monitoring, their concordance between the results of the clinical evaluations according to WHO criteria and the changes in the marker was 65%. The results presented here from serum CYFRA 21-1 are similar to those previously reported for various other markers used for monitoring of response to treatment (Hansen et al., 1980; Waalkes et al., 1980; Carney et al., 1982) in patients with SCLC. However, in 2 out of 14 patients in whom CYFRA 21-1 was measured at earlier points in addition to 4–8 weeks there was a decrease in CYFRA 21-1, indicating a faster excretion of this marker. We therefore recently conducted a prospective trial to monitor this marker. In this study CYFRA 21-1 is measured frequently during the early period after treatment (including day 1). From this trial we will be able to determine tumour lysis.

According to McKenzie et al. (1977) the practical value of a tumour marker depends on three factors: the frequency with which the marker is detected in the tumour population; the correlation between the blood level of the marker and the tumour burden; and the availability of effective treatment for the tumour. The findings of our study suggest that measurement of CYFRA 21-1 meets these criteria for a tumour marker of use in screening and planning treatment for NSCLC.

**Acknowledgements**

We thank Kiyoko Shiraishi, Yuri Umezawa and Kazuko Takahara for their assistance in data collection and statistical analysis. We also thank Drs Junji Shiraishi and Charles E Metz for providing the CLABROC program and the interpretation for the data. Furthermore, we gratefully acknowledge Dr Ralf Linke for critically reviewing the manuscript.

This work was supported in part by a Grant-in-Aid for Cancer Research from the Ministry of Health and Welfare (5S-1), and by a grant from Boehringer Mannheim.

**References**

AKOUN GM, SCARNA HM, MILLERON BJ, BENICHOU MP AND HERMAN DP. (1985). Serum neuron-specific enolase: a marker for disease extent and response for therapy to small-cell lung cancer. *Chest*, **87**, 39–43.

BORDENMÜLLER H, BANAUCH D, OFENLOCH B, JAWOREK D AND DESSAUER A. (1992). Technical evaluation of a new automated tumour marker assay: the Enzymun-Test® CYFRA 21-1. In *Tumor Associated Antigen, Oncogenes, Receptors, Cytokeratins in Tumor Diagnosis and Therapy at the Beginning of the Nineties*, Klapdor R (ed.) pp. 137–138. W Zuckshwerds: Munich.

BROERS JLV, KLEIN ROT M, OOSTENDORP T, HUYSMANS A, WAGENAAR SS, WIERSMA-VAN TILBURG AJM, VOOIJS GP AND RAMAEKERS FCS. (1987). Immunocytochemical detection of human lung cancer hereogeneity using antibodies to epithelial, neuronal and neurodendocrine antigens. *Cancer Res.*, **47**, 3225–3234.

BROERS JLV, RAMAEKERS FCS, KLEIN ROT M, OOSTENDORP T, HUYSMANS A, VAN MUIJEN GNP, WAGENARR SS AND VOOIJS GP. (1988). Cytokeratins in different types of human lung cancer as monitored by chain-specific monoclonal antibodies. *Cancer Res.*, **48**, 3221–3229.

CARNEY DN, IHDE DC, COHEN MH, MARANGOS PJ, BUNN PA, MINNA JD AND GAZDER AF. (1982). Serum neuron-specific enolase: a marker for disease extent and response to therapy of small-cell lung cancer (abstract). *Lancet*, **i**, 583–585.

EBERT W, LEICHTWEIS B, SCHAPOHLER B AND MULEY T. (1993). The new tumor marker CYFRA is superior to SCC antigen and CEA in the primary diagnosis of lung cancer. *Tumordiagn. u. Ther.*, **14**, 91–99.

GAAST AVD, SCHOENMAKERS CHH, KOK TC, BLIJENBERG BG, CORNILLIE F AND SPLINTER TAW. (1994). Evaluation of a new tumour marker in patients with non-small cell lung cancer: Cyfra 21.1. *Br. J. Cancer*, **69**, 525–528.

HANSEN M, HAMMER M AND HUMMER L. (1980). Diagnostic and therapeutic implications of ectopic hormone production in small cell carcinoma of the lung. *Thorac*, **35**, 101–106.

HOEFLER H AND DENK H. (1984). Immunohistochemical demonstration of cytokeratins in gastrointestinal carcinoids and their probable precursors cells. *Virchows Arch. B. Cell Pathol.*, **403**, 235–240.

KATO H AND TORIGOE T. (1977). Radioimmunoassay for tumor antigen of human cervical squamous cell carcinoma. *Cancer*, **40**, 1621–1628.

KAWAI T, OHKUBO S, HASEGAWA S, KURIYAMA T, KATO H, FUKUOKA M, YOSTUMOTO H, OHKAWA J AND KITAMURA S. (1993). Japanese multicenter evaluation of CYFRA in diagnosis and monitoring of primary lung cancer. *Proceedings of the 7th Hamburger Symposium uber Tumormarker*, p. 134.

KLAPDOR R. (1992). Arbeitsgruppe Qualitätskontrolle und Standardisierung von Tumormarkertests im Rahmen der Hamburger Symposien über Tumormarker. *Tumordigan. u Ther.*, **13**, XIX–XXII.

MATSUOKA Y, KUROKI M, KOGA Y, OGAWA H, NAKAZAWA N, TACHIBANA S AND MINAMIZAWA T. (1983). A new direct solid-phase radioimmunoassay for carcinoembryonic antigen without pretreatment of serum samples. *J. Immunol. Methods*, **58**, 31–47.

MCKENZIE CG, EVANS IMA, HILLYARD CJ, HILL P, CARTER S, TAN MK AND MACINTYRE, I. (1977). Biochemical markers in bronchial carcinoma. *Br. J. Cancer*, **36**, 700–707.

METZ CE, WANG P-L, KRONMAN HB. (1984). A new approach for testing the significance of differences between ROC curves measured from correlated data. In *Information Processing in Medical Imaging*, Deconinck F (ed.) pp. 432–445. Nijhoff: The Hague.

METZ CE, SHEN J-H, HERMAN BA. (1990). New methods for estimating a binormal ROC curve from continuously-distributed test results. Invited for presentation at the 1990 Joint Statistical Meetings of the American Statistical Society and the Biometric Society, Anaheim, CA, August.

MILLER AB, HOOGSTRATEN B, STAQUET M AND WINKLER A. (1981). Reporting results of cancer treatment. *Cancer*, **47**, 207–214.

MOLL R, FRANKE WW, SCHILLER DL, GEIGER B AND KREPLER R. (1982). The catalog of human cytokeratins: patterns of expression in normal epithelia, tumors and cultured cells. *Cell*, **31**, 11–24.

MOUNTAIN CF. (1986). A new international staging system for lung cancer. *Chest*, **89**, 225s–233s.

NICOLSON GL. (1987). Tumor cell instability, diversification, and progression to the metastatic phenotype: from oncogene to oncofetal expression. *Cancer Res.*, **47**, 1473–1487.

NOTOMI T, MORIKAWA J, KATO K, TSUCHIDA Y AND OHSAWA R. (1985). Radioimmunoassay development for human neuron-specific enolase: with some clinical results in lung cancers and neuroblastoma. *Tumor Biology*, **6**, 57–66.

OSBORN M AND WEBER K. (1982). Intermediate filaments: cell-type-specific markers in differentiation and pathology. *Cell*, **31**, 303–306.

PUJOL JL, GRENIER J, DAURES JP, DAVER A, PUJOL H AND MICHEL FB. (1993). Serum fragment of cytokeratin subunit 19 measured by CYFRA 21-1 immunoradiometric assay as a marker of lung cancer. *Cancer Res.*, **53**, 61–66.

PUJOL JL, SIMONY J, LAURENT JC, RICHER G, MARY H, BOUSQUET J, GODARD P AND MICHEL FB. (1989). Phentypic hererogeneity studied by immunohistochemistry and aneuploidy in non-small cell lung cancers. *Cancer Res.*, **49**, 2797–2802.

SACKETT DL, HAYNES B, GUYATT GH AND PETER T. (1991). The interpretation of diagnostic data. In *Clinical Epidemiology: a Basic Science for Clinical Medicine*, pp. 69–152. Little, Brown: Boston.

STIEBER P, HASHOLZNER U, BODENMULLER H, NAGEL D, SUNDER-PLASSMANN L, DIENEMANN H, MEIER W AND FATEH-MOGHADAM A. (1993). CYFRA21-1: a new marker in lung cancer. *Cancer*, **72**, 707–713.

WAALKES TP, ABELOFF MD, WOO KB, ETTINGER DS, RUDDON RW AND ALDENDERFER T. (1980). Carcinoembryonic antigen for monitoring patients with small cell carcinoma of the lung during treatment. *Cancer Res.*, **40**, 4420–4427.